# Supplementary material for: Depth-sensitive cerebral blood flow and low-frequency oscillations for consciousness assessment using time-domain diffuse correlation spectroscopy
Source: Neurophotonics. 2026 Mar 23;13(2):025005. doi: 10.1117/1.NPh.13.2.025005 (PMC13006803; doi:10.1117/1.NPh.13.2.025005)
Supplement: Supplementary file 1 [file NPh_013_025005_SD001.pdf]

Subject	Diagnosis	Age	Sex	Hemorrhage	Surgery	Resting-State (Fig. 3)	Smile Task (Fig. 4)	Notes
**H1–H25**	Healthy	21–39 (mean 26.9 ± 4.8)	14 M / 11 F	No	No	Yes	No	Resting-state cohort
**H26–H30**	Healthy	25–35 (mean 29.0 ± 5.5)	5 M	No	No	No	Yes	Smile-task cohort
**MCS-1**	MCS	40	M	No	No	Yes	Yes	Resting-state only
**MCS-2**	MCS	75	M	Yes (left temporal)	No	Yes	No	Resting-state only
**UWS-1**	UWS	40	M	Yes (multicompartment al)	Yes (decompressive craniectomy + skull reattachment)	No	Yes	Dual-probe bilateral + task
**Coma-1**	Coma	40	M	No	No	Yes	No	Resting-state only
**Coma-2**	Coma	40	M	No	No	Yes	No	Resting-state only
**Coma-3**	Coma	70	M	No	No	Yes	No	Resting-state only

Supplementary Table S1. Clinical characteristics and experimental inclusion of all subjects.

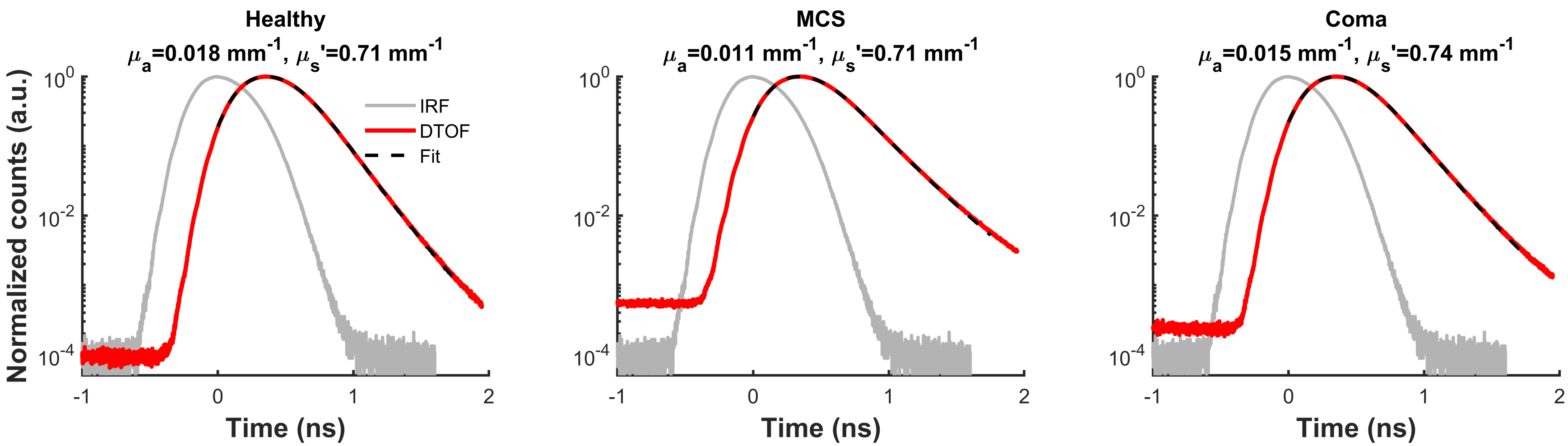

Supplementary Figure S1. Representative temporal point spread functions (TPSFs) and instrument response functions (IRFs) for a healthy subject, a minimally conscious state (MCS) patient with prior cranial surgery, and a comatose patient. Solid curves show measured DTOFs, and dashed curves show fits obtained using an analytical time-domain diffusion model assuming a semi-infinite homogeneous medium. The fitted absorption (μa) and reduced scattering (μs') coefficients represent effective bulk optical properties used as subject-specific inputs for TD-DCS blood-flow analysis and are not intended to reflect depth-resolved or layer-specific tissue properties.

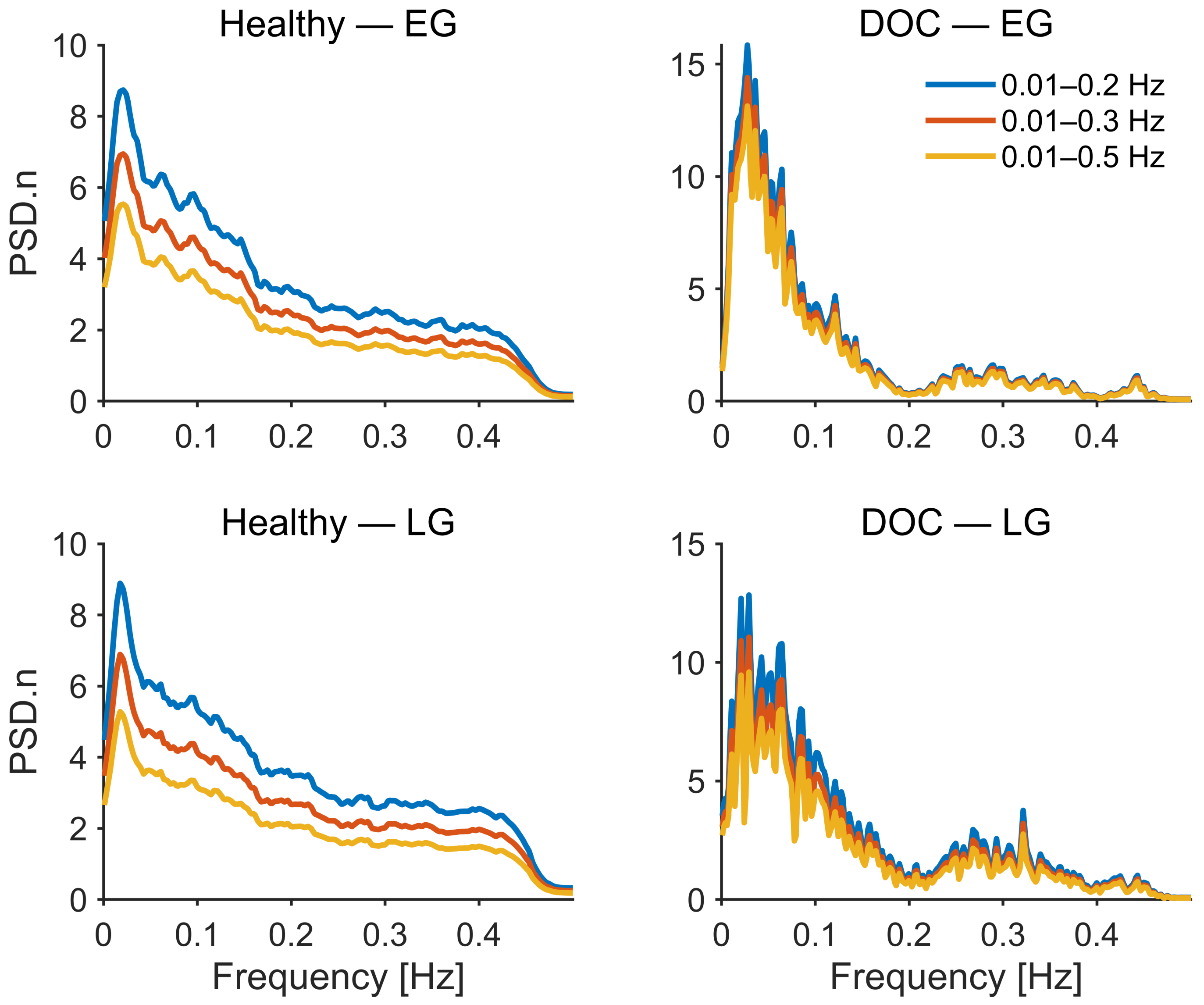

Supplementary Figure S2. Sensitivity analysis of normalized power spectral densities (PSD.n) to the choice of normalization bandwidth. Group-averaged PSD.n curves are shown for Healthy controls and patients with disorders of consciousness (DOC) for early-gate (EG) and late-gate (LG) data using three normalization ranges (0.01-0.2 Hz, 0.0-0.3 Hz, and 0.01-0.5 Hz). While the normalization bandwidth affects absolute scaling, the overall spectral shape and relative Healthy-DOC differences are preserved across conditions.
